# Increased Risk of Injury in Patients with Fabry Disease: A Nationwide Population-Based Cohort Study in Taiwan

**DOI:** 10.7150/ijms.120352

**Published:** 2026-05-29

**Authors:** Chun-Gu Cheng, Yu-Hsuan Chen, Wu-Chien Chien, Hung-Wen Chiu, Fei-Hung Hung, Hung-Pin Peng, Chi-Hsiang Chung, Chun-An Cheng

**Affiliations:** 1 Department of Emergency Medicine, Taoyuan Armed Forces General Hospital, Taoyuan, Taiwan.; 2 Department of Emergency Medicine, Tri-Service General Hospital, National Defense Medical University, Taipei, Taiwan.; 3 Department of Chest Medicine, Cheng-Hsin General Hospital, Taipei, Taiwan.; 4 School of Public Health, National Defense Medical University, Taipei, Taiwan.; 5 Department of Medical Research, Tri-Service General Hospital, National Defense Medical University, Taipei, Taiwan.; 6 Graduate Institute of Biomedical Informatics, Taipei Medical University, Taipei, Taiwan.; 7 Health Data Analytics and Statistics Center, Office of Data Science, Taipei Medical University, New Taipei City, Taiwan.; 8 Research Center of Data Science on Healthcare Industry, College of Management, Taipei Medical University, New Taipei City, Taiwan.; 9 Clinical Data Center, Office of Data Science, Taipei Medical University, New Taipei City, Taiwan.; 10 Department of Neurology, Tri-Service General Hospital, National Defense Medical University, Taipei, Taiwan.

**Keywords:** injury, Fabry disease, risk

## Abstract

**Background:**

Fabry disease (FD) is a lysosomal storage disorder leading to α-galactosidase A deficiency and glycosphingolipid accumulation. Neurological manifestations of FD, such as central nervous system involvement, peripheral neuropathy, and vestibular dysfunction that results in postural and cognitive impairment, may predispose individuals to injuries. However, the associations between the risk of injury and FD remains underexplored.

**Methods:**

Using the Taiwanese National Health Insurance Research Database, we conducted a nationwide cohort study of adult FD patients (International Classification of Diseases, Ninth Revision, Clinical Modification (ICD-9-CM) code 272.7)) who were diagnosed between 2001 and 2015. The comparison group was matched at a 1:4 ratio by age, sex, and index date. Injuries were identified via ICD-9-CM codes 800-999. Multivariate Cox proportional hazards models were applied to study the risk of injury, adjusting for sociodemographic factors and comorbidities.

**Results:**

The injury incidence rate was higher in the FD cohort (32.97/1,000 person-years) than in the control cohort (22.52/1,000 person-years). FD was significantly related to an increased risk of injury (adjusted hazard ratio: 1.642; 95% CI: 1.375-1.980; p < 0.001). FD patients had an 86% greater risk of motor vehicle traffic accidents and a 56% greater risk of falls than the control group. Higher risks of injury were also associated with the winter season, more complicated comorbidities, greater urbanization, and treatment at higher-level hospitals.

**Conclusion:**

This is the first large-scale study to demonstrate a significantly increased risk of injury among FD patients. These findings highlight the need for proactive injury risk assessments and tailored prevention strategies for this population. Future research should investigate the role of FD-related clinical phenotypes in injury susceptibility to enhance personalized care and improve outcomes.

## Introduction

Fabry disease (FD) is a rare X-linked lysosomal storage disorder characterized by deficient or absent activity of the enzyme α-galactosidase A (α-Gal A), which reduce the clearance of specific metabolites and thus subsequently cause cellular damage. The accumulation of globotriaosylceramide (Gb3) and related glycosphingolipids throughout the body results in the formation of a cascade of progressive, multisystemic pathologies involving the nervous, cardiovascular, renal, and gastrointestinal systems. Although FD was historically considered to predominantly affect males, it is now well recognized that heterozygous females may also develop significant disease manifestations, often with later onset and greater phenotypic variability 1. FD affects approximately 1 in 875 males in Taiwan, substantially more than in Western countries 2.

The clinical neurological features of FD, particularly small-fibre neuropathy and central nervous system involvement, result in impaired thermal and pain sensation, proprioceptive deficits, neuropsychiatric disturbances, and vestibular dysfunction 3-5. These impairments may compromise spatial awareness, motor coordination, and balance, thereby increasing patients' susceptibility to injury 6. Previous research has largely focused on organ-specific complications in various systems 1, with limited attention given to functional outcomes that may predispose patients to falls, trauma, or other injuries. Moreover, potential differences in injury susceptibility according to sex, disease phenotype, or multisystemic burden remain unclear. To date, no large-scale, population-based studies have investigated the incidence or determinants of injury in FD patients, and evidence-based preventive strategies are lacking.

We conducted a nationwide, population-based cohort study utilizing data from the Taiwan National Health Insurance Research Database to address this critical knowledge gap. Understanding the relationship between FD and the risk of injury is of particular clinical and public health importance in this setting.

We aimed to determine whether FD patients have a greater risk of injury compared to individuals in a matched control population. We hypothesized that FD patients would present a significantly greater incidence of injuries, because of the cumulative effects of multisystemic involvement. However, the common symptoms of FD can involve different types of injuries. Our findings can inform the development of tailored clinical surveillance strategies and preventive interventions to mitigate injury risk in this medically vulnerable population.

## Methods

Since 1995, the government in Taiwan has implemented a single-payer National Health Insurance (NHI) program covering all citizens. The Longitudinal Health Insurance Database includes 10% of the population and provides a representative sample of all NHI enrolees in terms of sex, age, and average insurance premiums according to the general population in Taiwan 7. Deidentified data from databases have been widely used in epidemiological and clinical research by Taiwanese scholars 8.

This study was approved by the Institutional Review Board of TSGHIRB A202305179 and was conducted in accordance with the principles outlined in the Declaration of Helsinki. Given the use of deidentified administrative claims data, the requirement for informed consent was waived.

**Figure 1:**
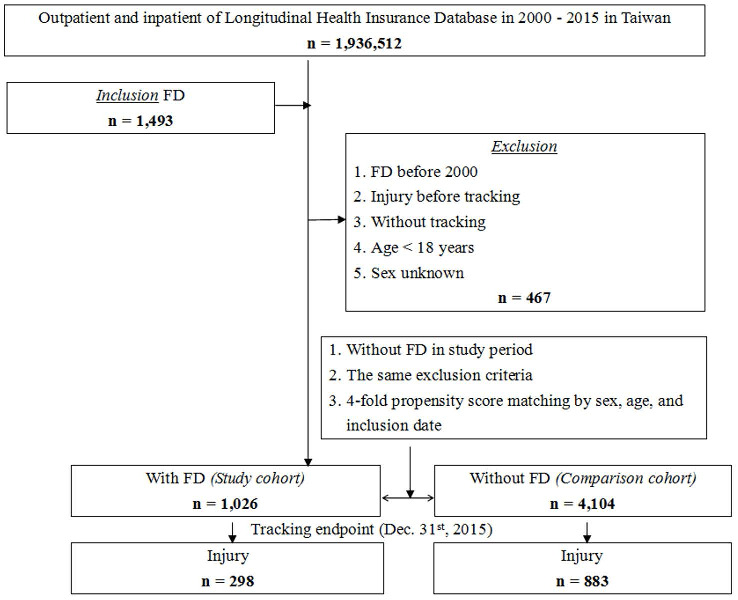
Flowchart of this study.

We identified patients aged 18 years or older who were newly diagnosed with FD between January 2001 and December 2015 according to the International Classification of Diseases, Ninth Revision, Clinical Modification (ICD-9-CM) code 272.7. Only patients with at least three outpatient visits or hospitalizations associated with the diagnosis of FD were included. Injury-related events were defined using the ICD-9-CM codes 800-999, and follow-up continued through December 31, 2015. A comparison cohort was identified from the remaining dataset of enrolees without any diagnosis of FD. The control group was matched at a 1:4 ratio according to age, sex, and index date to ensure comparability between FD patients and controls. A flowchart of the selection process is shown in Figure 1.

We also examined other possible risk factors, including other medical conditions identified by the ICD-9 CM code. FD patients receiving enzyme replacement treatment were identified on the basis of their prescription records. Additionally, we analysed external cause-of-injury codes (E-codes) to explore the mechanisms of injury. These mechanisms included unintentional injuries such as traffic accidents, poisoning, medically related incidents, falls, burns and fires, drowning, suffocation, crushing injuries, adverse drug reactions, other unintentional injuries), and intentional injuries (including suicide or homicide/abuse) (Table S1 shows the ICD-9-CM codes for comorbidities and the mechanisms of injury.).

The Charlson comorbidity index was revised to exclude hypertension, diabetes mellitus, renal disease, coronary artery disease, cerebrovascular disease, congestive heart failure, alcohol consumption, autoimmune disease, and malignancy (CCI_R).

Descriptive statistics were calculated, and continuous variables were analysed via Student's t test, whereas categorical variables were compared via the chi-square test. Multivariate Cox proportional hazards regression analysis was conducted to evaluate the association between FD and the risk of injury. The model was adjusted for potential confounding variables. The competing risk considered with all-cause mortality of FD was determined by Fine and Gray's competing risk model. Sensitivity analysis was performed by adding 1245 patients (including 249 with FD patients; 996 patients without FD) who experienced injury before tracking. Statistical significance was determined via two-tailed tests with a threshold of p ≤ 0.05. All analyses were performed via SPSS version 21.0.

## Results

**Figure 2:**
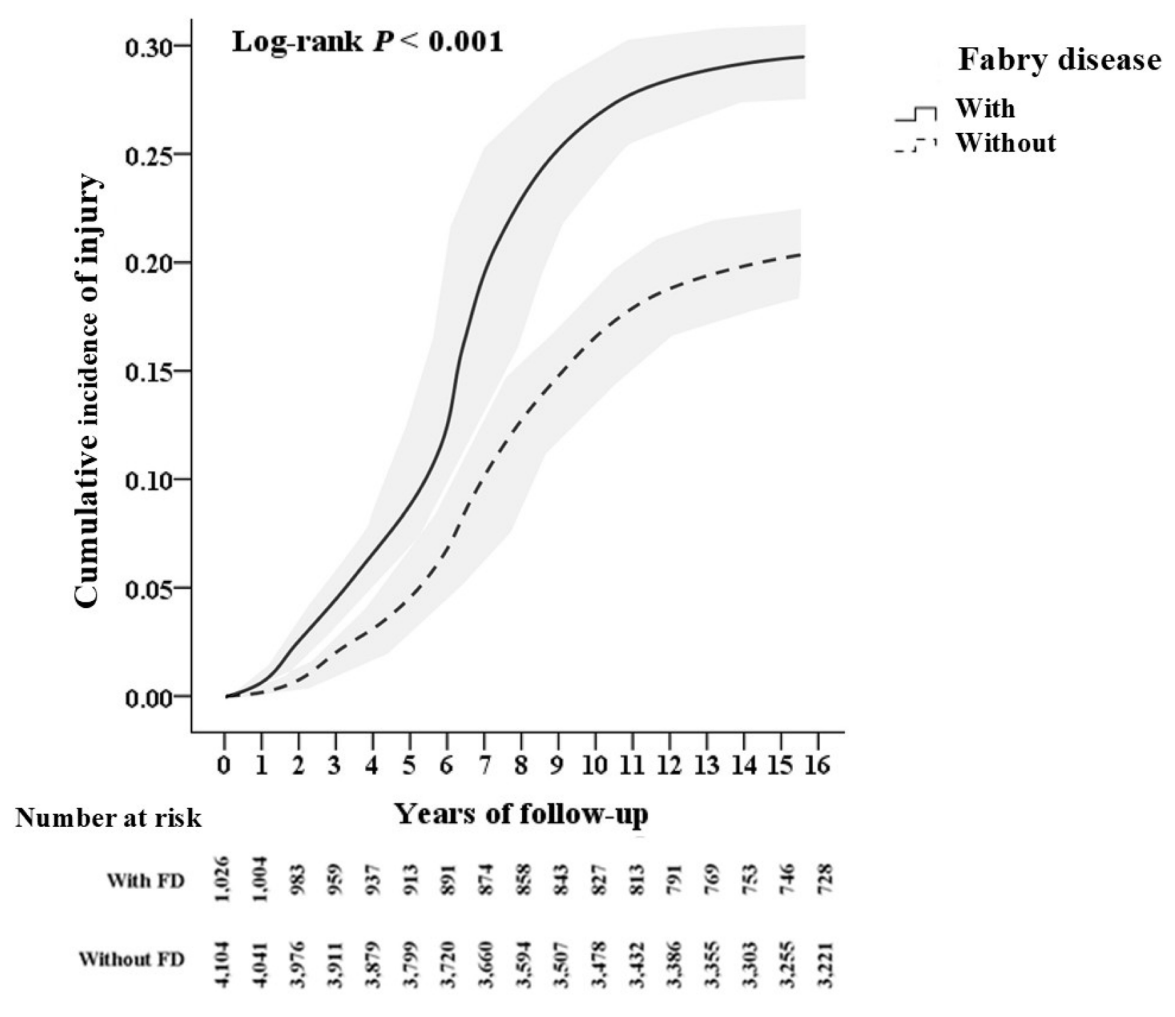
Kaplan-Meier curve for the cumulative incidence of injury. The patients were stratified on the basis of whether they had or did not have Fabry disease; a log-rank test showed *p*< 0.001. 1-year adjusted HR: 1.794 (95% CI: 0.762-2.732), *p* = 0.256. 2-year adjusted HR: 1.583 (95% CI: 1.101-2.248), *p* <0.001. 3-year adjusted HR: 1.679 (95% CI: 1.265-2.183), *p* < 0.001. 5-year adjusted HR: 1.657 (95% CI: 1.230-2.175), *p* < 0.001. > 5-year adjusted HR: 1.640 (95% CI: 1.371-1.976), *p* < 0.001.

This study included a total of 5,130 participants, comprising 1,026 FD patients and 4,104 matched controls. The incidence of injury was greater in the FD group, with a rate of 32.97 per 1,000 person-years, than in the control group, with a rate of 22.52 per 1,000 person-years, representing an absolute difference of 10.45 per 1,000 person-years (Figure 2). Based on the risk table shown in Figure 2, FD patients who had been diagnosed more than 1 year demonstrated a significantly higher risk of injury.

The baseline demographic characteristics and medical comorbidities of FD patients and control participants are summarized in Table 1. The FD patients demonstrated significantly higher rates of anxiety disorders, autoimmune diseases, and hyperthyroidism than the controls. Compared to the main analysis cohort, the sensitivity analysis cohort had a higher proportion of males; a younger mean age; higher rates of hypertension, hyperlipidaemia, depression, and anxiety; a higher proportion of individuals living in middle and eastern Taiwan; lower urbanization; a tendency to visit lower-level hospitals; and lower rates of osteoporosis and malignancy. (Table S2 shows a comparison between the main analysis and sensitivity analysis cohorts).

FD patients had a higher risk of injury than controls with an adjusted hazard ratio (HR) of 1.642 (95% CI: 1.375-1.98; p < 0.001). The adjusted HR of the competing risk model for mortality was1.684 (95% CI: 1.395-2.053; p < 0.001). The risk of injury was also higher in males than in females (adjusted HR: 1.301 (95% CI: 1.145-1.56, p < 0.001)). The risk of injury was also increased in young individuals (adjusted HR:2.03 (95% CI: 1.53-2.514; p < 0.001)) and middle-aged individuals (adjusted HR: 1.835 (95% CI: 1.321-2.308; p < 0.001)) compared to older individuals. Additional increases in the risks of injury were observed in individuals with hypertension (adjusted HR: 1.872 (95% CI: 1.318-2.243; p < 0.001)), diabetes mellitus (adjusted HR: 2.37 (95% CI: 1.682-2.557; p < 0.001)), hyperlipidaemia (adjusted HR: 1.732 (95% CI: 1.24-2.15; p < 0.001)), chronic kidney disease (adjusted HR: 1.685 (95% CI: 1.38-2.301; p < 0.001)), coronary artery disease (adjusted HR: 1.465 (95% CI:1.135-2,101; p < 0.001)), cerebrovascular disease (adjusted HR: 1.503(95% CI: 1.165-2.045; p < 0.001), depression (adjusted HR: 1.774 (95% CI: 1.489-2.056; p < 0.001); anxiety (adjusted HR: 1.732 (95% CI: 1.465-2.001; p < 0.001), alcohol-related disorder (adjusted HR: 2.462 (95% CI: 1.762-3.243; p < 0.001), autoimmune disease (adjusted HR: 1.765 (95% CI.: 1.224-2.508; p < 0.001), osteoporosis (adjusted HR: 1.312 (95% CI: 1.002-1.876; p=0.049), malignancy (adjusted HR: 1.503 (95% CI:1.324-1.98; p < 0.001), and migraine (adjusted HR: 1.264 (95% CI: 1.098-1.486; p = 0.002). The CCI_R was associated with injury with an adjusted HR of 1.165 (95% CI: 1.086-1.27; p = 0.007). Compared with spring, winter was associated with a greater risk of injuries, with an adjusted HR of 1.465 (95% CI: 1.113-1.774; p < 0.001) being reported. Higher urbanization level was associated with a greater risk of injury than the lowest urbanization level. Patients who visited medical centres exhibited a greater risk, with an adjusted HR 1.876 (95% CI: 1.444-2.223; p < 0.001) being observed; moreover, those who visited regional hospitals demonstrated a greater risk of injury with an adjusted HR 1.68 (95% CI: 1.206-2.01; p < 0.001) being observed than those who visited local hospitals. Sensitivity analysis revealed that FD was associated with the risk of injury with an adjusted HR of 1.689 (95% CI: 1.437-2.036; p < 0.001) (Table 2).

Stratified analyses demonstrated that, across all of the subgroups defined by age, sex, comorbidity burden, season, and urbanization level, FD was consistently associated with a significantly increased risk of subsequent injury. This association remained particularly pronounced among patients treated at medical centres and regional hospitals, where FD patients exhibited substantially higher injury risks than non-FD individuals did (Table S3 shows stratified analyses across all the subgroups). FD Patients were further stratified by clinical manifestations and treatment modalities, after which they were compared with non-FD patients. Those who presented with neuropathic symptoms, hypotension, syncope, sleep disorders, anaemia, and peripheral vertigo; those who were hospitalized; those who received enzyme replacement treatment; and those underwent rehabilitation demonstrated a markedly increased risk of injury. A longer duration of hospitalization was associated with a higher risk of injury. A longer duration of enzyme replacement treatment demonstrated lower risk of injury trend (Table S4 shows analyses stratified by clinical manifestations and treatment modalities, comparing individuals with and without FD). In terms of the different injury types, FD patients had increased risks of motor vehicle collision (HR: 1.863 (95% CI: 1.552-2.247, p < 0.001)), poisoning (HR: 2.207 (95% CI: 1.849-2.669, p < 0.001)), falls (HR: 1.56 (95% CI: 1.305-1.883, p < 0.001)), burns (HR: 1.213 (95% CI: 1.03-1.467, p = 0.035)), suffocation (HR: 1.46 (95% CI: 1.221-1.763, p < 0.001), crushing injuries (HR: 1.328 (95% CI: 1.11-1.604, p < 0.001)) and injuries caused by animals (HR: 1.217 (95% CI: 1.018-1.469, p = 0.041)). The severity of injuries was classified by using the injury severity score (ISS). The results demonstrated that major trauma (ISS ≥ 16) exhibited an adjusted HR of 2.018 (95% CI:1.69-2.434, p < 0.001), moderate trauma (ISS: 9-15) exhibited an adjusted HR of 1.958 (95% CI:1.64-2.361, p < 0.001) and minor trauma (ISS ≤ 8) exhibited an adjusted HR of 1.386 (95% CI: 1.161-1.671, p < 0.001). The more severe injuries demonstrated higher risks regarding traffic accidents, poisoning and fall (Table 3). A cross table of the different types of injuries and comorbid conditions was constructed (Table S5 shows the distribution of Fabry disease patients according to their comorbidities and causes of injury). Among individuals with FD-related neuropathy, 34.16% of traffic accidents and 14.29% of fall incidents were attributable to neuropathic manifestations. Among individuals with a history of stroke, 22.41% were involved in traffic accidents, and 15.52% experienced falls. Among individuals with a history of congestive heart failure, 25% experienced falls and traffic accidents. Among individuals with a history of coronary artery disease, 24.56% experienced traffic accidents, and 17.54% experienced falls. Among individuals with a history of peripheral vertigo, 19.05% experienced falls and traffic accidents. Among individuals with a history of hypotension, 32.65% experienced traffic accidents, and 24.49% experienced falls. Among individuals with a history of chronic kidney disease, 20.63% were involved in traffic accidents, and 11.11% experienced falls. However, among individuals with a history of sedation treatment, 17.33% were involved in traffic accidents and 13.33% experienced falls. The proportion of poisoning events was elevated among individuals receiving treatment for congestive heart failure (18/75%), sedation treatment (18.67%), neuropathy treatment (18.63%), and enzyme replacement therapy (12.36%).

## Discussion

This is the first epidemiological study to compare the incidence of injury between individuals with and without FD. Our findings revealed a significant association between FD and a 64% increase in the risk of injury. FD patients demonstrated an 86% greater risk of motor vehicle traffic accidents and a 56% greater risk of falls than matched controls.

### Neuropathy in FD

In FD, male patients tend to present with symptoms earlier than female patients do. Neurological involvement generally manifests around adolescence, followed by cardiac features and renal complications at thirty years of age 9. The underlying pathophysiology is thought to involve Gb3 accumulation in neural structures, including the perineurium, the endothelial cells of the vasa nervorum, the dorsal root ganglia, and the Schwann cells. Neuropathic pain predominantly affects the distal extremities and is characterized by diminished thermal sensation, burning dysaesthesias, and episodic pain crises that significantly impair quality of life and may alter gait patterns with a concomitant reduction in proprioception 3,10-11. Such neuromotor instability markedly increases the risk of falls and impairs limb coordination, and early physiotherapy is needed. FD patients exhibit increased risk of for various types of injury, primarily due to the fact that the sensory neuropathy resulting from the disease leads to impaired perception (especially in the extremities), thereby increasing the likelihood of burns and cuts from unnoticed contact with hot or sharp objects 10.

### Cognitive function in FD

Central nerve system involvement is well documented in FD patients 3. Imaging studies have demonstrated altered connectivity within the motor cortex and microstructural abnormalities in the thalamus, potentially implicating disrupted motor control pathways. Cerebellar involvement has also been reported 12. Changes in white matter are common in FD-related cognitive impairment 13,14. Emerging evidence has also linked FD to parkinsonian features with postural instability 15,16. Additionally, neuropsychiatric manifestations, including depression and anxiety, are common and may exacerbate the decline in cognitive function in FD patients 17, potentially increasing the incidence of injury. It might be beneficial to encourage aerobic exercise, mindfulness, and cognitive training to improve executive activity for driving and other daily activities. Furthermore, sedation treatment is associated with a reduced risk of injury.

### Stroke in FD

In lysosomal storage disorders such as FD, persistent antigenic stimulation perpetuates inflammatory signalling, rendering inflammation a chronic process 18. The pathological accumulation of Gb3 substrates within vascular endothelial cells is a hallmark feature of FD 19. This glycosphingolipid-induced vasculopathy disrupts normal vascular function, leading to impaired perfusion across multiple organ systems, including the renal, cardiac, central nervous, and peripheral nervous systems. In FD, the intima thickness increases, and flow-mediated dilation decreases 19. Reduced cerebral blood flow and cerebrovascular autoregulation impairment were noted in FD 20. An increased incidence of stroke in FD in the United States has been reported 21. FD is common in patients with cryptogenic stroke who present with proteinuria and basilar artery involvement 22. In Asian stroke populations, the prevalence of FD is approximately 0.62% according to the Fabry Outcome Survey, with male patients typically being affected at a younger age 23. Patients who have had a stroke present impaired cognitive function 24 and motor coordination. Consistent physiotherapy is needed to address for balance impairment and limb weakness. FD patients with comorbid stroke, neuropathy or chronic kidney disease were observed to have slower processing speed, and executive dysfunction, which can increase susceptibility to animal-related injuries. The caregivers of such patients need to prevent animal attacks.

### Cardiovascular conditions in FD

Lysosomal dysfunction in FD is related to impaired mitochondrial function and impaired cardiac energy metabolism 25. Gb3 accumulation promotes inflammation and fibrotic cascades in various tissues, including the myocardium, where it contributes to arrhythmias, left ventricular hypertrophy, interventricular septal thickening, myocardial fibrosis, and hypoxia. Additionally, mild to moderate valvular heart disease, particularly mitral and aortic valve thickening with mild regurgitation, is frequently observed 26, 27. Cardiac problems can increase falls 28. In patients with congestive heart failure, driving performance is reduced 29. Cardiovascular events can lead to symptoms such as dizziness, syncope, and cerebral hypoperfusion, all of which can impair driving performance 30.

### Chronic kidney disease in FD

Gb3 accumulation in the podocytes of the glomeruli within the nephrons results in fibrosis, tubular atrophy, and chronic kidney disease. Symptoms of FD include the involvement of various organ systems as a result of the accumulation of Gb3 in endothelial cells, initiating a downstream cascade of events leading to inflammation and organ damage 31. One-fifth of FD patients with chronic kidney disease experienced traffic accidents. Renal function impairment needs to be improved through treatment. Anaemia is common in FD patients, and its prevalence is strongly associated with declining renal function, the presence of heart failure and chronic inflammation. Anaemia may decrease the oxygen supply, resulting in impaired physical endurance and cognitive alertness, which may indirectly increase the risk of injury in this population 32. FD patients with anaemia demonstrate a higher risk of injury than those without anaemia. The cause of anaemia needs to be corrected as soon as possible.

### Dizziness in FD

Approximately eighty percent of FD patients reported problems of dizziness or balance. 5. Tinnitus is significantly more prevalent in individuals with FD, with an adjusted odds ratio of 1.5, than in the general population 33. The underlying pathophysiology may involve cochleovestibular ischaemia secondary to endothelial Gb3 accumulation. Persistent vestibular deficits may lead to spatial disorientation in FD patients, particularly during high-speed or curved motion 34, and these deficits can significantly impair postural control which is a function that is reliant on integrated visual, vestibular, and proprioceptive input 6. Among FD patients in China, 80% exhibit oculomotor problems and 40% demonstrate vestibulo-oculomotor dysfunctions 35. These impairments increase susceptibility to falls, traffic accidents, and injury and are often associated with psychological comorbidities such as anxiety and social withdrawal. Among individuals with a history of peripheral vertigo, one-fifth experienced falls or traffic accidents. Moreover, these individuals have required vestibular rehabilitation. Autonomic neuropathy is a recognized manifestation of FD and may contribute significantly to orthostatic hypotension, which presents as dizziness, lightheadedness, or syncope 36. Among FD patients with hypotension as a complication found one-third experienced traffic accidents and one-quarter experienced falls. Regular blood pressure checks are recommended, hypotension must be treated with inotropic agent.

### Sleep problems in FD

Approximately 65% of FD patients report of experiencing sleep disturbances, with a slightly higher prevalence among females. Excessive daytime sleepiness is disproportionately common in FD patients and may contribute to an increased risk of injury. In addition, the prevalence of obstructive and central sleep apnoea in FD patients appears comparable to that in the general population 37. Patients who have sleep problems associated with an increased risk of injury can reduce this risk by receiving sedation treatment to improve their sleep problems. Autonomic or central involvement can cause gastrointestinal dysmotility and, in severe cases, dysphagia, which may increase the risk of aspiration and suffocation 38,39.

### Other comorbidities of FD

Patients with diabetes mellitus have an increased rate of motor vehicle accidents 40. Our findings demonstrated that comorbidities, such as hypertension, hyperlipidaemia, and diabetes mellitus, are associated with an increased risk of injury. Fatigue and exercise intolerance are common symptoms caused by to cardiac and pulmonary dysfunctions in FD patients 41,42. Consistent with the findings of previous studies in young adults, alcohol-related disorders were associated with a significantly increased risk of injury 43. Migraines (which are characterized by photophobia, altered sensory processing, and central nerve system dysfunction) are known to impair driving performance with an HR of 1.78 being reported for traumatic head injury 44,45 and an odds ratio of 3.3 being reported for motor vehicle collisions within one year 46. In our study, alcohol-related disorders caused by complications related to the alcohol consumption were associated with an increased risk of injury. Moreover, exposure to low ambient temperatures has been shown to increase the risk of falls 47. Consistent with these findings, our study revealed an increased risk of injury during the winter.

### Treatment of FD

In Taiwan, enzyme replacement treatment and pharmacological chaperones are covered by health insurance but require rigorous clinical documentation and approval, including evidence of characteristic symptoms (e.g., small-fibre neuropathy, left ventricular hypertrophy, and proteinuria) and, in some cases, pathological confirmation via biopsy 48. Treatment helps mitigate symptoms and improve quality of life but cannot provide a cure 49. FD patients who received enzyme replacement treatment tended to have more severe symptoms and a greater risk of injury than those who did not receive treatment. Impaired renal and hepatic function further predisposes FD patients to drug toxicity and poisoning; however, adverse reactions to enzyme replacement therapy may temporarily reduce physical or cognitive alertness 17. An increased proportion of poisoning events were observed among FD patients receiving treatment for congestive heart failure, as well as sedation treatment, neuropathy treatment, and enzyme replacement therapy. Before treatment, renal and hepatic function need to be checked. Adjunctive rehabilitation strategies tailored to FD patients may further support balance, coordination, and mobility 50, but FD patients seem to have an increased risk of injury, potentially because of delayed interventions.

### Risk regarding short duration of FD and small cells of different type injuries

The non-significant hazard ratio observed at the 1-year follow-up (p = 0.256) may indicate that injury risk in patients with FD progressively accumulates with disease advancement. During the early stages, subclinical organ involvement and compensatory physiological mechanisms may mitigate overt clinical manifestations, thereby attenuating detectable risk and diluting short-term effect estimates. These findings underscore the importance of longitudinal evaluation and suggest that extended follow-up is necessary to more accurately delineate the dynamic and evolving risk profile associated with FD. The distribution of patients with FD across comorbid conditions revealed extremely sparse event counts in certain categories, including burns (n = 3), animal-related injuries (n = 1), and cutting/piercing injuries (n = 6). Given the limited number of cases detected in these subgroups, these observations should be interpreted with caution. Accordingly, the analyses pertaining to these categories are considered to be exploratory in nature to avoid over-interpretation.

The strengths of this study include the use of a large, nationwide population-based dataset. The inclusion of individuals who participate in Taiwan's universal health insurance system (which is characterized by low copayments and comprehensive coverage) minimized selection bias. The increased injury risk in FD has implications for healthcare utilization, disability prevention, and resource allocation, potentially leading to hospitalization, prolonged rehabilitation, and a socioeconomic burden. Additionally, the NHI claims data capture all healthcare utilization events for FD patients and injuries across the entire population, reducing the recall bias inherent in self-reported data. The genetic diagnosis of FD was partially funded by the Rare Disease Foundation in Taiwan, and treatment is fully covered by Taiwan's single-payer health insurance, reducing the financial burden and ensuring timely medical care. With highly accessible healthcare, patients who experience injuries can receive timely medical assistance. Annual evaluations should assess visual, auditory, cognitive, and motor functions, and driving should be discouraged to reduce the risk of accidents when deficits are detected. Early identification of high-risk individuals could enable timely interventions. It is recommended that FD patients undergo a 10-metre walk test every year, and those with a reduced gait speed < 0.8 metre/second should receive a walking aid and early physiotherapy to prevent falls 51, 52. FD patients are recommended to undergo a clock drawing test to evaluate their cognitive activity, executive activity and hand coordination. Clinicians should advise neuropathic pain management, vestibular rehabilitation, and avoidance of high-risk activities. Enhancing awareness among caregivers, employers, social services, and public health agencies is essential for creating safer environments and reducing injury-related morbidity. These findings underscore the need for multidisciplinary approaches involving neurology, rehabilitation, cardiology, and public health professionals.

### Limitations

Several limitations of this study should be acknowledged. First, the dataset lacked information on disease severity, genetic variants, laboratory parameters (such as nerve conduction study, echocardiography, renal function test, and haemoglobin test results), anthropometric data (such as height and weight), and lifestyle factors, including smoking and alcohol consumption (all of which may influence gait stability and injury risk). Registry studies can overcome this limitation. Second, the administrative claims data did not capture detailed information of the severity of nerve injury, renal function, and cardiac function regarding the mechanisms, thereby limiting the granularity of injury characterization and precluding a full understanding of the injury spectrum in FD patients. The integration of richer clinical datasets and the inclusion of family members or healthcare providers are needed to enhance the comprehensiveness of FD risk profiling and injury prevention strategies. Third, although comorbidities were observed to be associated with increases in FD patients' rates of higher traffic accidents, falls and poisoning events, the cause-effect relationship could not be established in this study. Future studies must be designed to survey the actual risk. Fourth, the generalizability of our findings is limited, as the majority of participants were of Han Chinese ethnicity. The clinical burden of FD in Taiwan is thus disproportionately high and warrants targeted public health and clinical research efforts 2. The support of the national health insurance system ensured access to high-quality care while minimizing the economic burden on patients. Injured patients received better care because of improved access to healthcare services. Medical centres specializing in FD provided professional diagnosis and comprehensive care, including genetic screening for family members, which facilitated early detection. Future research should aim to incorporate more diverse populations and healthcare systems. Fifth, our incidence information was derived from the injury codes included in the claim dataset; thus, some minor injuries that were resolved without outpatient or inpatient visits may have gone unreported, thereby causing the incidence of injury to be underestimated.

## Conclusion

This study revealed a significantly increased risk of injuries among FD patients. The progressive multisystem involvement characteristic of FD may contribute to impaired gait stability, altered cognitive function, and reduced execution, all of which predispose patients to accidental harm. Given these findings, clinicians should remain vigilant in monitoring the evolving clinical course of FD and proactively assessing injury risk. Moreover, caregivers should provide appropriate support in daily activities to help mitigate the occurrence of injuries in this vulnerable population.

## Supplementary Material

Supplementary tables.

## Figures and Tables

**Table 1:** Baseline characteristics of the study participants.

	Fabry disease (1,026)	No Fabry disease (4,104)	*p*	SMD	Effect Sizes (Cohen's d) 95%CI
Male	380 (37.04%)	1,520 (37.04%)	0.999	0.0347	0.000 (-0.068, 0.068)
Age (years)	41.18 ± 22.45	41.20 ± 22.49	0.978	0.0349	-0.001 (-0.069, 0.068)
Hypertension	165 (16.08%)	633 (15.42%)	0.603	0.0347	-0.018 (-0.086, 0.050)
Diabetes mellitus	210 (20.47%)	809 (19.71%)	0.588	0.0349	-0.019 (-0.087, 0.050)
Hyperlipidaemia	124 (12.09%)	481 (11.72%)	0.745	0.0349	-0.011 (-0.080, 0.057)
Chronic kidney disease	139 (13.55%)	577 (14.06%)	0.672	0.0349	0.015 (-0.054, 0.083)
Coronary artery disease	179 (17.45%)	702 (17.11%)	0.796	0.0347	-0.009 (-0.077, 0.059)
Cerebrovascular disease	186 (18.13%)	711 (17.32%)	0.544	0.0347	-0.021 (-0.089, 0.047)
Congestive heart failure	102 (9.04%)	365 (8.17%)	0.342	0.0349	-0.031 (-0.100, 0.037)
Depression	130 (12.67%)	488 (11.89%)	0.493	0.0349	-0.024 (-0.092, 0.045)
Anxiety	124 (12.09%)	325 (7.92%)	< 0.001*	0.0349	-0.139 (-0.208, -0.071)
Alcohol-related disorder	223 (21.73%)	911 (22.20%)	0.749	0.0349	0.011 (-0.057, 0.080)
Autoimmune disease	67 (6.53%)	203 (4.95%)	0.042*	0.0349	-0.068 (-0.137, 0.000)
Osteoporosis	55 (5.36%)	219 (5.34%)	0.975	0.0347	-0.001 (-0.069, 0.067)
Malignancy	141 (13.74%)	578 (14.08%)	0.778	0.0349	0.010 (-0.059, 0.078)
Migraine	109 (10.62%)	412 (10.04%)	0.579	0.0349	-0.019 (-0.088, 0.049)
Hyperthyroidism	66 (6.43%)	193 (4.70%)	0.024*	0.0349	-0.076 (-0.144, -0.007)
CCI_R	0.84 ± 1.02	0.81 ± 1.00	0.392	0.0349	0.030 (-0.039, 0.098)
Season			0.869	0.0349	-0.028 (-0.096, 0.041)
Spring (Mar - May)	265 (25.83%)	1,011 (24.63%)			
Summer (Jun - Aug)	281 (27.39%)	1,125 (27.41%)			
Autumn (Sep - Nov)	243 (23.68%)	991 (24.15%)			
Winter (Dec - Feb)	237 (23.10%)	977 (23.81%)			
Location			< 0.001*	0.0352	-0.456 (-0.525, -0.387)
Northern Taiwan	503 (49.03%)	1,312 (31.97%)			
Middle Taiwan	262 (25.54%)	1,017 (24.78%)			
Southern Taiwan	209 (20.37%)	1,068 (26.02%)			
Eastern Taiwan	52 (5.07%)	513 (12.50%)			
Outlying islands	0	194 (4.73%)			
Urbanization level			< 0.001*	0.0352	-0.391 (-0.460, -0.322)
1 (The highest)	351 (34.21%)	1,084 (26.41%)			
2	482 (46.98%)	1,316 (32.07%)			
3	77 (7.50%)	775 (18.88%)			
4 (The lowest)	116 (11.31%)	929 (22.64%)			
Level of healthcare			< 0.001*	0.0367	-1.210 (-1.282, -1.138)
Medical centre	899 (87.62%)	1,314 (32.02%)			
Regional hospital	126 (12.28%)	1,368 (33.33%)			
Local hospital	1 (0.10%)	1,422 (34.65%)			

SMD = standardized mean difference

**Table 2:** Risk factors for injury according to Cox regression.

Variables	Adjusted hazard ratio	*p*
Fabry disease		
Non-competing risk model	1.642(95% CI:1.375-1.980)	< 0.001*
Competing risk model	1.684 (95% CI: 1.395-2.053)	< 0.001*
Sensitivity analysis	1.689 (95% CI: 1.437-2.036)	< 0.001*
Male	1.301 (95% CI: 1.145-1.560)	< 0.001*
Age group		
Young	2.03 (95% CI: 1.53-2.514)	< 0.001*
Middle-aged	1.835 (95% CI: 1.321-2.038)	< 0.001*
Older	Reference	
Hypertension	1.872 (95% CI: 1.318-2.243)	< 0.001*
Diabetes mellitus	2.37 (95% CI: 1.682-2.557)	< 0.001*
Hyperlipidaemia	1.732 (95% CI: 1.24-2.15)	< 0.001*
Chronic kidney disease	1.685 (95% CI: 1.38-2.301)	< 0.001*
Coronary artery disease	1.465 (95% CI: 1.135-2.101)	< 0.001*
Cerebrovascular disease	1.503(95% CI: 1.165-2.045)	< 0.001*
Congestive heart failure	1.432 (95% CI: 0.998-2.713)	0.052
Depression	1.774 (95% CI: 1.489-2.056)	< 0.001*
Anxiety	1.732 (95% CI: 1.465-2.001)	< 0.001*
Alcohol-related disorder	2.462 (95% CI: 1.762-3.243)	< 0.001*
Autoimmune disease	1.765 (95% CI: 1.224-2.508)	< 0.001*
Osteoporosis	1.312 (95% CI: 1.002-1.876)	0.049*
Malignancy	1.503 (95% CI:1.324-1.98)	< 0.001*
Migraine	1.264 (95% CI: 1.098-1.486)	0.002*
Hyperthyroidism	1.087 (95% CI: 0.865-1.271)	0.145
CCI_R	1.165 (95% CI: 1.086-1.27)	0.007*
**Season**		
Spring	Reference	
Summer	1.065 (95% CI: 0.725-1.33)	0.259
Autumn	1.267 (95% CI: 0.973-1.589)	0.077
Winter	1.465 (95% CI: 1.113-1.774)	< 0.001*
Urbanization level		
1 (The highest)	1.597 (95% CI: 1.331-1.806)	< 0.001*
2	1.552 (95% CI: 1.328-1.794)	< 0.001*
3	1.303 (95% CI: 1.114-1.52)	< 0.001*
4 (The lowest)	Reference	
Level of healthcare		
Medical centre	1.876 (95% CI: 1.444-2.223)	< 0.001*
Regional hospital	1.68 (95% CI: 1.206-2.01)	< 0.001*
Local hospital	Reference	

CI: confidence interval; CCI_R: Charlson comorbidity index revised to exclude hypertension, diabetes mellitus, chronic kidney disease, coronary artery disease, cerebrovascular disease, congestive heart failure, alcohol consumption, autoimmune disease, and malignancy. Sensitivity analysis included patients with a previous history of injury (Fabry disease, n = 249; no Fabry disease, n = 996)

**Table 3:** Risk of injury subgroups by Cox regression

Fabry disease	With			Without *(reference)*					
Injury subgroups	Events	PYs	Rate	Events	PYs	Rate	aHR (95% CI)	*p*	Related conditions
Overall	298	9,039.08	32.97	883	39,201.92	22.52	1.642 (1.375-1.980)	< 0.001*	
Unintentional injury	246	9,039.08	27.22	681	39,201.92	17.37	1.758 (1.47-2.124)	< 0.001*	
Traffic injuries	132	9,039.08	14.60	345	39,201.92	8.80	1.863 (1.552-2.247)	< 0.001*	N, ERT, Hypo, CAD
Major trauma (ISS≥ 16)	26	9,039.08	2.88	53	39,201.92	1.35	2.387 (1.999-2.878)	< 0.001*	
Moderate trauma (ISS: 9 - 15)	44	9,039.08	4.87	96	39,201.92	2.45	2.230 (1.867-2.691)	< 0.001*	
Minor trauma (ISS ≤ 8)	62	9,039.08	6.86	196	39,201.92	5.00	1.539 (1.273-1.857)	< 0.001*	
Poisoning	39	9,039.08	4.31	86	39,201.92	2.19	2.207 (1.849-2.669)	< 0.001*	N, Sed, ERT
Major trauma (ISS≥ 16)	9	9,039.08	1.00	16	39,201.92	0.41	2.994 (2.503-3.612)	< 0.001*	
Moderate trauma (ISS: 9 - 15)	13	9,039.08	1.44	27	39,201.92	0.69	2.435 (2.035-2.939)	< 0.001*	
Minor trauma (ISS ≤ 8)	17	9,039.08	1.88	43	39,201.92	1.10	1.888 (1.582-2.271)	< 0.001*	
Falls	43	9,039.08	4.76	134	39,201.92	3.42	1.56 (1.305-1.883)	< 0.001*	N, ERT, Hypo, Sed, CAD, Stroke
Major trauma (ISS≥ 16)	10	9,039.08	1.11	29	39,201.92	0.74	1.946 (1.631-2.348)	< 0.001*	
Moderate trauma (ISS: 9 - 15)	15	9,039.08	1.66	44	39,201.92	1.12	1.840 (1.541-2.220)	< 0.001*	
Minor trauma (ISS≤ 8)	18	9,039.08	1.99	61	39,201.92	1.56	1.327 (1.112-1.607)	< 0.001*	
Burns and fires	3	9,039.08	0.33	12	39,201.92	0.31	1.213 (1.03-1.467)	0.035*	N, Stroke, CKD
Drowning	2	9,039.08	0.22	9	39,201.92	0.23	1.083 (0.905-1.306)	0.118	
Suffocation	9	9,039.08	1.00	30	39,201.92	0.77	1.46 (1.221-1.763)	< 0.001*	Sed, CHF,PV
Crushing / cutting / piercing	6	9,039.08	0.66	22	39,201.92	0.56	1.328 (1.11-1.604)	< 0.001*	ERT, CKD, Sed, CAD, Hypo
Excessive heat	0	9,039.08	0.00	1	39,201.92	0.03	0	0.999	
Injury caused by animal	1	9,039.08	0.11	4	39,201.92	0.10	1.217 (1.018-1.469)	0.041*	N, Stroke, CKD
Electric current injury	0	9,039.08	0.00	2	39,201.92	0.05	0	0.999	
Other unintentional injuries	11	9,039.08	1.22	36	39,201.92	0.92	1.483 (1.241-1.792)	< 0.001*	
Intentional injury	23	9,039.08	2.54	101	39,201.92	2.58	1.108 (0.925-1.337)	0.104	
Suicide	13	9,039.08	1.44	59	39,201.92	1.51	1.071 (0.896-1.29)	0.139	
Homicide / abuse	10	9,039.08	1.11	42	39,201.92	1.07	1.159 (0.97-1.398)	0.073	
Intention unknown	5	9,039.08	0.55	18	39,201.92	0.46	1.352 (1.134-1.622)	< 0.001*	
Without E-code	24	9,039.08	2.66	83	39,201.92	2.12	1.413 (1.182-1.705)	< 0.001*	
Major trauma (ISS≥ 16)	56	9,039.08	6.20	135	39,201.92	3.44	2.018 (1.69-2.434)	< 0.001*	
Moderate trauma (ISS: 9 - 15)	99	9,039.08	10.95	246	39,201.92	6.28	1.958 (1.64-2.361)	< 0.001*	
Minor trauma (ISS≤ 8)	143	9,039.08	15.82	502	39,201.92	12.81	1.386 (1.161-1.671)	< 0.001*	

*p<0.05; PYs: person-years; rate: per 1,000 PYs; CI: confidence interval; ISS: injury severity score; aHR: adjusted hazard ratio, adjusted for the variables listed in Table 2.; N: neuropathy; Hypo: hypotension; CAD: coronary artery disease; Sed: sedation treatment; CKD: chronic kidney disease; CHF: congestive heart failure; PV: peripheral vertigo
