# Supplementary material for: Increased Risk of Injury in Patients with Fabry Disease: A Nationwide Population-Based Cohort Study in Taiwan
Source: Int J Med Sci. 2026 May 29;23(7):2376–86. doi: 10.7150/ijms.120352 (PMC13280734; doi:10.7150/ijms.120352)
Supplement: Supplementary file 1 — Supplementary tables. [file ijmsv23p2376s1.pdf]

Table S1. The International Classification of Diseases, Ninth Revision, Clinical Modification code of the comorbidities and mechanisms of injury.

	**ICD-9-CM code**
Comorbidities	
Hypertension	401 - 405
Diabetes mellitus	250
Hyperlipidemia	272, excluding 272.4
Chronic kidney disease	580 - 589
Coronary artery disease	410 - 414
Cerebrovascular accident	430 - 438
Congestive heart failure	428
Depression	296.2, 296.3, 296.82, 330.4, 311
Anxiety	300.1 - 300.3, 300.5 - 300.9
Alcohol-related disorder	291, 303, 305.0, 571.0 - 571.4
Autoimmune disorders	710, 714
Osteoporosis	733.0
Malignancy	140 - 209
Migraine	346
Hyperthyroidism	242
Neuropathy	357.89, 357.9, 729.2
Autonomic neuropathy	337
Hypotension	458
Syncope	780.2
Sleep disorders	307.4, 327, 780.5
Anemia	285
Peripheral vertigo	386.0, 386.11, 386.12, 386.8, 386.19, 386.9
Events	
Injury	800 - 999
Different type of injury by E code	
Unintentional injury	E800 - E949
Traffic injuries	E800 - E849
Poisoning	E850 - E869
Falls	E880 - E888
Burns and fires	E890 - E899
Drowning	E910
Suffocation	E911 - E915

Crushing / cutting / piercing	E916 - E920
Excessive heat	E900
Injury caused by animal	E906
Electric current injury	E925
Other unintentional injuries	E870 - E879, E901 - E905, E907 - E909, E921 - E924, E926 - E949
Intentional injury	E950 - E979, E990 - E999
Suicide	E950 - E959
Homicide / abuse	E960 - E969
Intention unknown	E980 - E989
Treatment	
Physiotherapy	OP91.1-OP93.6
Occupational therapy	OP93.83
Enzyme replacement therapy	ATC: A16AB03, A16AB04
Sedation	ATC: N05C

ICD-9-CM code: International Classification of Diseases, Ninth Revision, Clinical Modification code; ATC: anatomical therapeutic code

Table S2. The comparison between the main analysis and sensitivity analysis cohorts

**Injury history**	**Without (**5,130)	**With (**1,245)	***p***
Male	1,900 (37.04%)	672 (53.98%)	< 0.001*
Age (years)	41.20 ± 22.48	35.06 ± 19.68	< 0.001*
Hypertension	798 (15.56%)	246 (19.76%)	< 0.001*
Diabetes mellitus	1,019 (19.86%)	275 (22.09%)	0.080
Hyperlipidemia	605 (11.79%)	198 (15.90%)	< 0.001*
Chronic kidney disease	716 (13.96%)	164 (13.17%)	0.472
Coronary artery disease	881 (17.17%)	189 (15.18%)	0.091
Cerebrovascular disease	897 (17.49%)	191 (15.34%)	0.071
Congestive heart failure	467 (8.34%)	118 (9.48%)	0.196
Depression	618 (12.05%)	193 (15.50%)	0.001*
Anxiety	449 (8.75%)	172 (13.82%)	< 0.001*
Alcohol-related disorder	1,134 (22.11%)	284 (22.81%)	0.591
Autoimmune disease	270 (5.26%)	55 (4.42%)	0.224
Osteoporosis	274 (5.34%)	42 (3.37%)	0.004*
Malignancy	719 (14.02%)	121 (9.72%)	< 0.001*
Migraine	521 (10.61%)	106 (8.51%)	0.081
Hyperthyroidism	259 (5.05%)	62 (4.98%)	0.921
CCI_R	0.82 ± 1.00	0.78 ± 0.94	0.200
Season			0.711
Spring (Mar - May)	1,276 (24.87%)	315 (25.30%)	
Summer (Jun - Aug)	1,460 (27.41%)	322 (25.86%)	
Autumn (Sep - Nov)	1,234 (24.05%)	301 (24.18%)	
Winter (Dec - Feb)	1,214 (23.66%)	307 (24.66%)	
Location			< 0.001*
Northern Taiwan	1,815 (35.38%)	394 (31.65%)	
Middle Taiwan	1,279 (24.93%)	320 (25.70%)	
Southern Taiwan	1,277 (24.89%)	285 (22.89%)	
Eastern Taiwan	565 (11.01%)	171 (13.73%)	
Outlets islands	194 (3.78%)	75 (6.02%)	
Urbanization level			< 0.001*
1 (The highest)	1,435 (27.97%)	335 (26.91%)	
2	1,798 (35.05%)	406 (32.61%)	
3	852 (16.61%)	283 (22.73%)	
4 (The lowest)	1,045 (20.37%)	221 (17.75%)	
Level of healthcare			< 0.001*

Medical center	2,213 (43.14%)	295 (23.69%)
Regional hospital	1,494 (29.12%)	503 (44.01%)
Local hospital	1,423 (27.74%)	447 (35.90%)

*p<0.05

Table S3. Factors of injury stratified by variables listed in the table by using Cox regression

**Fabry disease**	**With**			**Without** *(Reference)*				
**Stratified**	**Events**	**PYs**	**Rate**	**Events**	**PYs**	**Rate**	**aHR (95% CI)**	***p***
**Overall**	298	9,039.08	32.97	883	39,201.92	22.52	1.642 (1.375-1.98)	< 0.001*
**Sex**								
Male	119	3,349.20	35.53	330	14,519.32	22.73	1.757 (1.468-2.125)	< 0.001*
Female	179	5,689.88	31.46	553	24,682.60	22.40	1.573 (1.306-1.894)	< 0.001*
**Age group (yrs)**								
18 - 44	272	7,920.25	34.34	776	34,263.71	22.65	1.702 (1.426-2.053)	< 0.001*
45 - 64	24	995.53	24.11	97	4,298.64	22.57	1.199 (1.005-1.446)	0.047*
≧ 65	2	123.30	16.22	10	639.57	15.64	1.163 (0.972-1.398)	0.074
**Hypertension**								
Without	240	7,503.11	31.99	741	33,069.48	22.41	1.594 (1.322-1.904)	< 0.001*
With	58	1,535.97	37.76	142	6,132.44	23.16	1.833 (1.537-2.209)	< 0.001*
**Diabetes mellitus**								
Without	230	7,080.87	32.48	705	31,426.59	22.43	1.621 (1.365-1.95)	< 0.001*
With	68	1,958.21	34.73	178	7,775.33	22.89	1.708 (1.442-2.067)	< 0.001*
**Hyperlipidemia**								
Without	259	7,902.14	32.78	776	34,492.90	22.50	1.634 (1.325-1.903)	< 0.001*
With	39	1,136.94	34.30	107	4,709.02	22.72	1.704 (1.428-2.059)	< 0.001*
**Chronic kidney disease**								
Without	235	7,518.05	31.26	753	33,632.19	22.39	1.566 (1.311-1.888)	< 0.001*
With	63	1,521.03	41.42	130	5,569.73	23.34	1.996 (1.668-2.405)	< 0.001*
**Coronary artery disease**								
Without	241	7,418.04	32.49	729	32,458.11	22.46	1.62 (1.342-1.942)	< 0.001*
With	57	1,621.04	35.16	154	6,743.81	22.84	1.777 (1.552-2.184)	< 0.001*
**Cerebrovascular accident**								
Without	240	7,356.93	32.62	724	32,305.62	22.41	1.630 (1.352-1.942)	< 0.001*
With	58	1,682.15	34.48	159	6,896.30	23.06	1.688 (1.412-2.124)	< 0.001*
**Congestive heart failure**								
Without	266	8,113.97	32.78	803	35,686.11	22.50	1.631 (1.352-1.924)	< 0.001*
With	32	925.11	34.59	80	3,515.81	22.75	1.707 (1.43-2.068)	< 0.001*
**Depression**								
Without	256	7,856.64	32.58	776	34,498.64	22.49	1.621 (1.355-1.972)	< 0.001*
With	42	1,182.44	35.52	107	4,703.28	22.75	1.786 (1.479-2.13)	< 0.001*
**Anxiety**								

Without	256	7,921.14	32.32	809	36,059.33	22.44	1.61 (1.342-1.93)	< 0.001*
With	42	1,117.94	37.57	74	3,142.59	23.55	1.791 (1.498-2.159)	< 0.001*
**Alcohol-related disorder**								
Without	228	7,075.56	32.22	682	30,433.42	22.41	1.612 (1.321-1.947)	< 0.001*
With	70	1,963.52	35.65	201	8,768.50	22.92	1.792 (1.482-2.188)	< 0.001*
**Autoimmune disease**								
Without	275	8,420.34	32.66	837	37,228.30	22.48	1.630 (1.365-1.962)	< 0.001*
With	23	618.74	37.17	46	1,973.62	23.31	1.794 (1.525-2.214)	< 0.001*
**Osteoporosis**								
Without	279	8,519.18	32.75	834	37,076.19	22.49	1.631 (1.36-1.964)	< 0.001*
With	19	519.90	36.55	49	2,125.73	23.05	1.780 (1.425-2.188)	< 0.001*
**Malignancy**								
Without	253	7,726.24	32.75	755	33,633.02	22.45	1.670 (1.325-1.927)	< 0.001*
With	45	1,312.84	34.28	128	5,568.90	22.98	1.782 (1.425-2.08)	< 0.001*
**Migraine**								
Without	265	8,059.20	32.88	792	35,204.27	22.50	1.640 (1.373-1.975)	< 0.001*
With	33	979.88	33.68	91	3,997.65	22.76	1.662 (1.392-2.003)	< 0.001*
**Hyperthyroidism**								
Without	276	8,439.75	32.70	839	37,334.45	22.47	1.620 (1.351-1.957)	< 0.001*
With	22	599.33	36.71	44	1,867.47	23.56	1.784 (1.428-2.183)	< 0.001*
**Season**								
Spring	67	2,220.14	30.18	206	9,523.74	21.63	1.552 (1.304-1.874)	< 0.001*
Summer	77	2,449.91	31.43	229	10,364.28	22.10	1.597 (1.338-1.925)	< 0.001*
Autumn	68	2,026.03	33.56	218	9,427.85	23.12	1.630 (1.368-1.974)	< 0.001*
Winter	86	2,343.00	36.71	230	9,886.05	23.27	1.778 (1.492-2.137)	< 0.001*
**Urbanization level**								
1 (The highest)	90	2,563.27	35.11	224	9,801.45	22.85	1.725 (1.445-2.083)	< 0.001*
2	105	3,118.09	33.67	285	12,651.14	22.53	1.679 (1.405-2.023)	< 0.001*
3	37	1,127.30	32.82	177	7,897.23	22.41	1.643 (1.377-1.982)	< 0.001*
4 (The lowest)	66	2,230.42	29.59	197	8,852.10	22.25	1.482 (1.21-1.793)	< 0.001*
**Level of healthcare**								
Hospital center	136	3,532.91	38.50	277	11,854.31	23.37	1.859 (1.562-2.239)	< 0.001*
Regional hospital	97	2,937.10	33.03	325	14,309.25	22.71	1.63 (1.365-1.964)	< 0.001*
Local hospital	65	2,569.07	25.30	281	13,038.36	21.55	1.317 (1.009-1.589)	0.046*

*p<0.05; PYs: person-years; rate: per 1,000 PYs; CI: confidence interval; aHR: adjusted hazard ratio: risk adjusted for the variables listed in Table 2.

Interaction (Fabry disease x Age group), p = 0.004

Table S4. Factors of injury among different Fabry disease subgroups by using Cox regression

Fabry disease subgroups	Population	Events	PYs	Rate	Adjusted HR (95% CI)	*p*
Without FD	4,104	883	39,201.92	22.52	Reference	
With FD	1,026	298	9,039.08	32.97	1.642 (1.375–1.980)	< 0.001*
With FD, outpatient	617	145	5,426.49	26.72	1.327 (1.114–1.603)	< 0.001*
With FD, inpatient length of day: 1 - 7	305	100	2,753.04	36.32	1.886 (1.529–2.194)	< 0.001*
With FD, inpatient length of day: > 7	104	53	859.55	61.66	3.071 (2.586–3.794)	< 0.001*
With FD, without enzyme replacement therapy	723	209	6,370.75	32.81	1.634 (1.368–1.970)	< 0.001*
With FD, with ERT: < 3 months	97	30	853.87	35.13	1.750 (1.465–2.110)	< 0.001*
With FD, with ERT: ≥ 3 months, < 1 year	166	48	1,467.58	32.71	1.628 (1.364–1.964)	< 0.001*
With FD, with ERT: ≥ 1 year	40	11	346.88	31.71	1.570 (1.321–1.908)	< 0.001*
With FD, with enzyme replacement therapy	303	89	2,668.33	33.35	1.661 (1.391–2.003)	< 0.001*
With FD, without rehabilitation therapy	651	187	5,733.91	32.61	1.624 (1.360–1.959)	< 0.001*
With FD, with rehabilitation therapy	375	111	3,305.17	33.58	1.673 (1.401–2.017)	< 0.001*
With FD, without neuropathy	502	137	4,418.94	31.00	1.544 (1.293–1.862)	< 0.001*
With FD, with neuropathy	524	161	4,620.14	34.85	1.736 (1.453–2.093)	< 0.001*
With FD, without autonomic neuropathy	1,013	295	8,928.81	33.04	1.646 (1.378–1.984)	< 0.001*
With FD, with autonomic neuropathy	13	3	110.27	27.21	1.355 (1.135–1.634)	< 0.001*
With FD, without hypotension	895	249	7,889.80	31.56	1.572 (1.316–1.895)	< 0.001*
With FD, with hypotension	131	49	1,149.28	42.64	2.123 (1.778–2.561)	< 0.001*
With FD, without syncope	1,004	290	8,846.81	32.78	1.633 (1.367–1.969)	< 0.001*
With FD, with syncope	22	8	192.27	41.61	2.072 (1.735–2.499)	< 0.001
With FD, without sleep disorders	561	152	5,003.90	30.38	1.513 (1.267–1.824)	< 0.001*
With FD, with sleep disorders	465	146	4,035.18	36.18	1.802 (1.509–2.173)	< 0.001*
With FD, without anemia	837	241	7,373.86	32.68	1.628 (1.363–1.963)	< 0.001*
With FD, with anemia	189	57	1,665.22	34.23	1.705 (1.428–2.056)	< 0.001*
With FD, without peripheral vertigo	901	256	7,938.42	32.25	1.606 (1.342-1.935)	< 0.001*
With FD, with peripheral vertigo	125	42	1,100.66	38.16	1.903 (1.594-2.296)	< 0.001*
With FD, without sedation	759	223	6,689.37	33.34	1.662 (1.392-2.004)	< 0.001*
With FD, with sedation	267	75	2,349.71	31.92	1.588 (1.328-1.915)	< 0.001*

PYs: Person-years; PYs: person-years; Rate: per 1,000 PYs; CI: confidence interval; ERT: enzyme replacement treatment; adjusted HR: Adjusted hazard ratio, adjusted for the variables listed in Table 2.

Table S5. Distribution of patients with Fabry disease by comorbidities conditions and causes of injury

	Fabry disease (FD) with comorbidities										
Causes of injury (cases)	CKD	CAD	Stroke	CHF	ERT	Neuropathy	Hypotension	Syncope	PV	Sedation	***p***
Overall	63	57	58	32	89	161	49	8	42	75	
Traffic injuries (132)	13 (20.63%)	14 (24.56%)	13 (22.41%)	8 (25.00%)	20 (22.47%)	55 (34.16%)	16 (32.65%)	2 (25.00%)	8 (19.05%)	13 (17.33%)	< 0.001*
Poisoning (39)	6 (9.52%)	5 (8.77%)	7 (12.07%)	6 (18.75%)	11 (12.36%)	30 (18.63%)	4 (8.16%)	1 (12.50%)	2 (4.76%)	14 (18.67%)	< 0.001*
Falls (43)	7 (11.11%)	10 (17.54%)	9 (15.52%)	8 (25.00%)	16 (17.98%)	23 (14.29%)	12 (24.49%)	4 (50.00%)	8 (19.05%)	10 (13.33%)	< 0.001*
Burns and fires (3)	2 (3.17%)	0 (0.00%)	2 (3.45%)	0 (0.00%)	1 (1.12%)	2 (1.24%)	1 (2.04%)	0 (0.00%)	2 (4.76%)	0 (0.00%)	0.425
Crushing / cutting / piercing (6)	5 (7.94%)	4 (7.02%)	2 (3.45%)	3 (9.38%)	5 (5.62%)	3 (1.86%)	4 (8.16%)	0 (0.00%)	3 (7.14%)	5 (6.67%)	0.183
Injury caused by animal (1)	1 (1.59%)	0 (0.00%)	1 (1.72%)	0 (0.00%)	0 (0.00%)	1 (0.62%)	0 (0.00%)	0 (0.00%)	0 (0.00%)	0 (0.00%)	0.769

CKD: chronic kidney disease; CAD: coronary artery disease; CVA: cerebrovascular accident; CHF: congestive heart failure; ERT: enzyme replacement therapy; PV: peripheral vertigo
